# Supplementary material for: Pyruvate Dehydrogenase Kinase 1 inhibition mediated oxidative phosphorylation enhancement in cartilage promotes osteoarthritis progression
Source: BMC Musculoskelet Disord. 2023 Jul 20;24:597. doi: 10.1186/s12891-023-06585-6 (PMC10357736; doi:10.1186/s12891-023-06585-6)
Supplement: Supplementary file 1 — Additional file 1. [file 12891_2023_6585_MOESM1_ESM.zip › MF.Gsea.1653627316242/GOMF_CAMP_BINDING.html]

Details for gene set GOMF\_CAMP\_BINDING[GSEA]

|  || Dataset | 55457\_collapsed\_to\_symbols.cls.cls #OA\_versus\_normal.cls.cls #OA\_versus\_normal\_repos |
| Phenotype | cls.cls#OA\_versus\_normal\_repos |
| Upregulated in class | normal |
| GeneSet | GOMF\_CAMP\_BINDING |
| Enrichment Score (ES) | -0.52129424 |
| Normalized Enrichment Score (NES) | -1.512969 |
| Nominal p-value | 0.036395147 |
| FDR q-value | 0.17221913 |
| FWER p-Value | 1.0 |
Table: GSEA Results Summary

  

Fig 1: Enrichment plot: GOMF\_CAMP\_BINDING      
 Profile of the Running ES Score & Positions of GeneSet Members on the Rank Ordered List

  

| SYMBOL | TITLE | RANK IN GENE LIST | RANK METRIC SCORE | RUNNING ES | CORE ENRICHMENT || 1 | POPDC3 | popeye domain containing 3 [Source:HGNC Symbol;Acc:HGNC:17649] | 2235 | 0.335 | -0.0999 | No |
| 2 | CNGB1 | cyclic nucleotide gated channel subunit beta 1 [Source:HGNC Symbol;Acc:HGNC:2151] | 4509 | 0.112 | -0.2517 | No |
| 3 | POPDC2 | popeye domain containing 2 [Source:HGNC Symbol;Acc:HGNC:17648] | 5578 | 0.026 | -0.3289 | No |
| 4 | HCN4 | hyperpolarization activated cyclic nucleotide gated potassium channel 4 [Source:HGNC Symbol;Acc:HGNC:16882] | 5590 | 0.025 | -0.3243 | No |
| 5 | PRKAR1B | protein kinase cAMP-dependent type I regulatory subunit beta [Source:HGNC Symbol;Acc:HGNC:9390] | 5806 | 0.008 | -0.3392 | No |
| 6 | PRKAR2B | protein kinase cAMP-dependent type II regulatory subunit beta [Source:HGNC Symbol;Acc:HGNC:9392] | 7034 | -0.082 | -0.4164 | No |
| 7 | HCN2 | hyperpolarization activated cyclic nucleotide gated potassium and sodium channel 2 [Source:HGNC Symbol;Acc:HGNC:4846] | 8387 | -0.185 | -0.4808 | Yes |
| 8 | PDE10A | phosphodiesterase 10A [Source:HGNC Symbol;Acc:HGNC:8772] | 8477 | -0.192 | -0.4455 | Yes |
| 9 | PDE4D | phosphodiesterase 4D [Source:HGNC Symbol;Acc:HGNC:8783] | 8797 | -0.216 | -0.4228 | Yes |
| 10 | PDE2A | phosphodiesterase 2A [Source:HGNC Symbol;Acc:HGNC:8777] | 8872 | -0.223 | -0.3796 | Yes |
| 11 | PDE4A | phosphodiesterase 4A [Source:HGNC Symbol;Acc:HGNC:8780] | 9957 | -0.317 | -0.3942 | Yes |
| 12 | PRKAR1A | protein kinase cAMP-dependent type I regulatory subunit alpha [Source:HGNC Symbol;Acc:HGNC:9388] | 10093 | -0.330 | -0.3321 | Yes |
| 13 | PRKAR2A | protein kinase cAMP-dependent type II regulatory subunit alpha [Source:HGNC Symbol;Acc:HGNC:9391] | 10664 | -0.389 | -0.2909 | Yes |
| 14 | RAPGEF2 | Rap guanine nucleotide exchange factor 2 [Source:HGNC Symbol;Acc:HGNC:16854] | 11041 | -0.435 | -0.2244 | Yes |
| 15 | RAPGEF3 | Rap guanine nucleotide exchange factor 3 [Source:HGNC Symbol;Acc:HGNC:16629] | 11380 | -0.483 | -0.1446 | Yes |
| 16 | RAPGEF4 | Rap guanine nucleotide exchange factor 4 [Source:HGNC Symbol;Acc:HGNC:16626] | 11963 | -0.586 | -0.0611 | Yes |
| 17 | PDE4B | phosphodiesterase 4B [Source:HGNC Symbol;Acc:HGNC:8781] | 12057 | -0.609 | 0.0654 | Yes |
Table: GSEA details [plain text format]

  

Fig 2: GOMF\_CAMP\_BINDING      
 Blue-Pink O' Gram in the Space of the Analyzed GeneSet

  

Fig 3: GOMF\_CAMP\_BINDING: Random ES distribution      
 Gene set null distribution of ES for **GOMF\_CAMP\_BINDING**
